# Genome-Wide Bimolecular Fluorescence Complementation-Based Proteomic Analysis of *Toxoplasma gondii* ROP18’s Human Interactome Shows Its Key Role in Regulation of Cell Immunity and Apoptosis

**DOI:** 10.3389/fimmu.2018.00061

**Published:** 2018-02-05

**Authors:** Jing Xia, Ling Kong, Li-Juan Zhou, Shui-Zhen Wu, Li-Jie Yao, Cheng He, Cynthia Y. He, Hong-Juan Peng

**Affiliations:** ^1^Department of Pathogen Biology, Guangdong Provincial Key Laboratory of Tropical Disease Research, School of Public Health, Southern Medical University, Guangzhou, China; ^2^Department of Biological Sciences, National University of Singapore, Singapore, Singapore

**Keywords:** *Toxoplasma gondii*, ROP18, human interactome, bimolecular fluorescence complementation, genome-wide

## Abstract

*Toxoplasma gondii* rhoptry protein ROP18 (*Tg*ROP18) is a key virulence factor secreted into the host cell during invasion, where it modulates the host cell response by interacting with its host targets. However, only a few *Tg*ROP18 targets have been identified. In this study, we applied a high-throughput protein–protein interaction (PPI) screening in human cells using bimolecular fluorescence complementation (BiFC) to identify the targets of Type I strain ROP18 (ROP18_I_) and Type II strain ROP18 (ROP18_II_). From a pool of more than 18,000 human proteins, 492 and 141 proteins were identified as the targets of ROP18_I_ and ROP18_II_, respectively. Gene ontology, search tool for the retrieval of interacting genes/proteins PPI network, and Ingenuity pathway analyses revealed that the majority of these proteins were associated with immune response and apoptosis. This indicates a key role of *Tg*ROP18 in manipulating host’s immunity and cell apoptosis, which might contribute to the immune escape and successful parasitism of the parasite. Among the proteins identified, the immunity-related proteins N-myc and STAT interactor, IL20RB, IL21, ubiquitin C, and vimentin and the apoptosis-related protein P2RX1 were further verified as ROP18_I_ targets by sensitized emission-fluorescence resonance energy transfer (SE-FRET) and co-immunoprecipitation. Our study substantially contributes to the current limited knowledge on human targets of *Tg*ROP18 and provides a novel tool to investigate the function of parasite effectors in human cells.

## Introduction

*Toxoplasma gondii* is an obligate intracellular protozoon that causes zoonotic toxoplasmosis. It is estimated that one third of the world’s population is chronically infected with this parasite (1). *T. gondii* belongs to the phylum of Apicomplexa, characterized by the presence of an apical complex containing secretory organelles, including rhoptries, micronemes, and dense granules (2). Rhoptry discharges a family of proteins termed rhoptry proteins (ROPs) that are of importance for host cell invasion, intracellular survival, and interference with host functions (3, 4).

*T. gondii* isolates collected from North America and Europe primarily fall into one of the three distinct clonal lineages, types I, II, and III (5), which present a number of different phenotypes, such as growth, migration, and transmigration (6). The best characterized phenotype is their virulence in laboratory mice (7, 8): Type I strains exhibit acute lethal virulence [lethal dose (LD_100_) ≈ 1], whereas types II and III strains are much less virulent [median LD_50_ ≥ 10^5^] (9, 10). According to previous forward genetic mapping studies, in which Types I, II, or III were intercrossed to identify the virulence determinant genes, the highly polymorphic *rop18* gene was identified as a key virulence determinant (11, 12). *Tg*ROP18 is a serine/threonine kinase secreted from the rhoptry into the parasitophorous vacuole membrane (PVM) and host cytosol during parasite invasion (13), of which the Type I strain (ROP18_I_) (RH strain, GenBank accession NO: AFO54817.1) and the Type II strain (ROP18_II_) (ME49, GenBank accession NO: XP_002367757.1) are different at 28 amino acid sites.

In murine cells, ROP18_I_ can target and inactivate the immunity-related GTPases (IRGs) Irga6 and Irgb6 by phosphorylating a critical threonine residue in the switch loop 1 of the IRGs, thereby disrupting their accumulation on the PVM and protecting the parasites from destruction (14, 15). Although the precise molecular functions of *Tg*ROP18 in human cells remain obscure, it is known that it regulates parasite’s multiplication in human cells and host cell apoptosis. It has been reported that a Type III strain (CEP) expressing ROP18_I_ showed a dramatic increase in replication rate in human foreskin fibroblasts (HFFs) in comparison to the wild-type CEP strain without *Tg*ROP18 expression (13). It has also been shown that ROP18_I_ inhibits cell apoptosis *via* the mitochondrial apoptosis pathway in human embryonic kidney 293 T cells (16). *Tg*ROP18 exerts its regulation on some important host cell signaling by interacting with its host targets. For instance, ROP18_I_ phosphorylates and mediates the degradation of the host endoplasmic reticulum (c)-bound transcription factor ATF6β, which is expressed in both human and murine cells, resulting in compromised CD8^+^ T cell-mediated host defense against *T. gondii* infection (17). ROP18_I_ has also been shown to associate with p65, a member of the human NF-κB family of transcription factors, and targets this protein for ubiquitin-dependent degradation to suppress the human NF-κB pathway (18). Despite the important roles of the virulence factor *Tg*ROP18 in disrupting host cell functions and preserving survival of parasites in human cells, only a few binding partners of ROP18_I_ have been determined, and the complex regulatory network of protein–protein interactions (PPIs) between ROP18_I_ and host cell proteins remains to be elucidated. Moreover, very little is known regarding the host targets of ROP18_II_, even though it is functionally expressed in Type II strains and is capable of conferring virulence to a Type III strain (11, 19).

**Figure 1:**
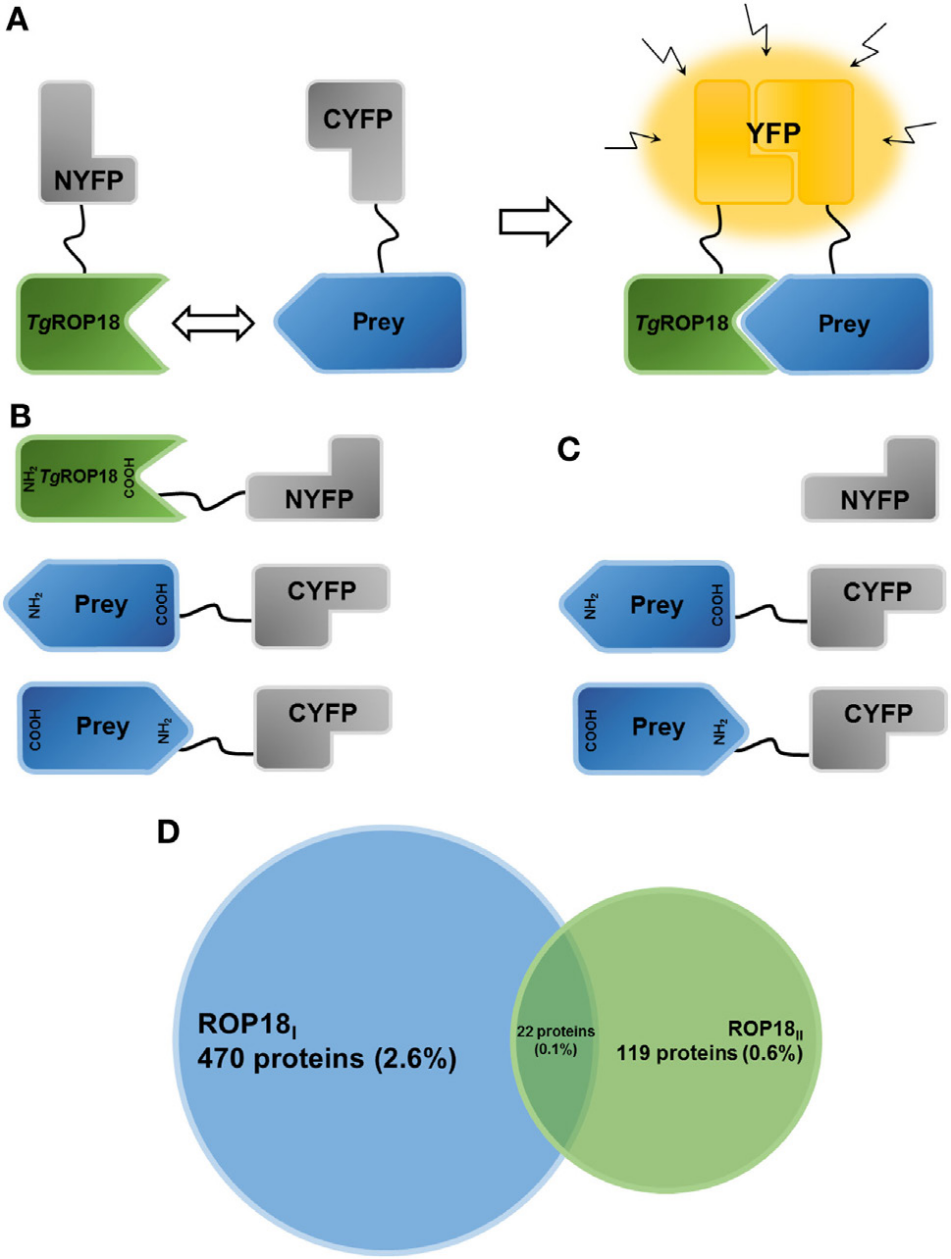
Establishment of the HT-BiFC screening system and *Tg*ROP18-interacting proteins. **(A)** Principle of the bimolecular fluorescence complementation (BiFC) assay. The non-fluorescent fragments of a fluorescent reporter protein are fused with the proteins of interest and expressed in human cells. If the interaction between the proteins of interest takes place, the split fragments will be pulled close enough to refold together and reconstitute the functional fluorescent entity. **(B)** Schematic representation illustrating the *Tg*ROP18/prey BiFC constructs generated in the present study. *Tg*ROP18 is fused with the N-terminal fragment of YFP (NYFP) at the C-terminus, and the prey protein is tethered with the C-terminal fragment of YFP (CYFP) at either the N- or C-terminus. **(C)** Schematic representation illustrating the control screening. Non-fused NYFP is mated with each CYFP-prey/prey-CYFP constructs. **(D)** Venn diagram depicting the number (percentage) of ROP18_I_-specific targets (blue), ROP18_II_-specific targets (green), and ROP18_I_/ROP18_II_ targets (in the middle).

A previous study has identified eight *Tg*ROP18-interacting proteins with a yeast two-hybrid (YTH) system (20). However, YTH generates a high occurrence of false positives and requires that the interacting proteins accumulate in the yeast nucleus (21). More recently, by using a protein array approach, Yang et al. have identified 68 substrates of the *Tg*ROP18 kinase, and four of them have been validated as the host targets (22). Although protein array is useful for comprehensive screens of protein functions, it requires pure functional proteins that are difficult to obtain because of the difficulties in expressing the proteins in a soluble form with correct folding (23). The bimolecular fluorescence complementation (BiFC) technique has been proven to be a useful and efficient tool to study PPIs. The BiFC assay is based on the principle that two non-fluorescent fragments [e.g., amino-yellow fluorescence protein, NYFP, or carboxyl-yellow fluorescence protein, C-terminal fragment of YFP (CYFP)] of a fluorescent reporter protein (e.g., yellow fluorescence protein, YFP) can refold together and reconstitute the functional fluorescent entity when they are in close proximity, for example by fusing to a pair of interacting proteins (Figure 1A) (24). Thus, the fluorescence intensity is proportional to the amount of formed dimer and can be detected by microscopy or flow cytometry (25). Here, we applied a high-throughput PPI screening based on BiFC (HT-BiFC) combined with a Gateway cloning system (26) to identify the potential *Tg*ROP18 (ROP18_I_ and ROP18_II_) interaction partners within a human ORFeome library containing more than 18,000 human cDNA clones (27). This screening helped us gain insights into the biological functions of *Tg*ROP18 in human cells.

## Materials and Methods

### Parasites and Cell Lines

The RH and PRU strains of *T. gondii* were maintained by serial passage in HFFs, as described previously (28). The HFFs (#ATCC SCRC-1041), Phoenix (#ATCC CRL-3213), and COS-7 (#ATCC CRL-1651) cell lines were purchased from the American Type Culture Collection (Manassas, VA, USA). The HTC75 cell line was kindly provided by Professor Wenbin Ma (Sun Yat-Sen University, Guangzhou, China). Parasites and cells were cultured in Dulbecco’s Modified Eagle Medium (DMEM, Gibco, #11995065) supplemented with 10% fetal bovine serum (Gibco, #16000044) and 1% penicillin/streptomycin (Gibco, #15070063) at 37°C in a 5% CO_2_ incubator.

### Antibodies

Anti-NMI rabbit monoclonal antibody (#183724) was obtained from Abcam (Cambridge, MA, USA). Anti-FLAG mouse monoclonal antibody (#AE005) was obtained from Abclonal (Woburn, MA, USA). Anti-HA rabbit monoclonal (#3724) and anti-β-Actin rabbit monoclonal (#4970) antibodies were obtained from Cell Signaling Technology (Danvers, MA, USA). Normal rabbit control IgG (#AB-105-C) was obtained from R&D Systems (Minneapolis, MN, USA). Anti-P2RX1 goat polyclonal (#sc-31491) and normal goat IgG (#sc-2028) antibodies were obtained from Santa Cruz Biotechnology (Dallas, TX, USA). Normal mouse IgG (#12-371) was obtained from Sigma-Aldrich (Billerica, MA, USA). Mono- and polyubiquitinylated conjugates monoclonal (FK2) antibody (#BML-PW8810) was obtained from Enzo Life Sciences (Farmingdale, NY, USA).

### Plasmid Construction

Total RNA of *T. gondii* RH and PRU tachyzoites was extracted using the RNeasy Plus Mini Kit (#74034, Qiagen, Germantown, MD, USA) following manufacturer’s instructions. The cDNA fragments of ROP18_I_ (ToxoDB #TGGT1_205250) and ROP18_II_ (ToxoDB #TGME49_205250) were amplified by RT-PCR from the total RNA of the RH and PRU tachyzoites with the forward primer 5′-ATAGCGGCCGCAATGTTTTCGGTACAGCG-3′ and the reverse primer 5′-GGCGCGCCCTTCTGTGTGGAGATG-3′. The cDNAs of ROP18_I_ and ROP18_II_ were then fused with the N-terminal fragment (residues 1–155) of yellow fluorescent protein (NYFP) at the C-terminus to construct the bait vectors, pBabe-CMV-ROP18_I_-NYFP-neo and pBabe-CMV-ROP18_II_-NYFP-neo, respectively (Figure 1B). The cDNAs of N-myc and STAT interactor (NMI), interleukin 20 receptor-β (IL20RB), purinergic receptor P2X1 (P2RX1), interleukin 21 (IL21), ubiquitin C (UBC), and vimentin were individually amplified by PCR from the human ORFeome v3.1 (Open Biosystems) and subcloned into pcDNA3.1 for eukaryotic expression, or into pEYFP-C1 for expression fused with enhanced yellow fluorescent protein. In addition, ROP18_I_ and ROP18_II_ cDNAs were, respectively, subcloned into pcDNA3.1 for eukaryotic expression, and into pECFP-N1 for expression fused with enhanced cyan fluorescent protein. All constructs were verified by DNA sequencing.

### HT-BiFC Assay

The HT-BiFC screening was conducted by Longjie Biotechnology Co., Ltd. (Foshan, Guangdong, China). Bait vectors were transfected into the packaging cell lines, Phoenix cells, to generate the retrovirus, and the harvested retroviruses were used to infect HTC75 cells. Stable bait cell lines expressing ROP18_I_-NYFP or ROP18_II_-NYFP were obtained after 10 days of selection with 300 μg/mL G418. Meanwhile, a pool of prey vectors were constructed from the human ORFeome v7.1 library, containing 18,414 human open reading frames (ORFs), using the Gateway recombination system. At the end of the process, 17,076 colonies with a coverage of 93% of all human ORFs were successfully obtained (27). The prey collections were tethered to the C-terminal fragment (residues 156–239) of YFP (CYFP) at either the N- or C-terminus (pCL-CMV-prey-CYFP-puro and pCL-CMV-CYFP-prey-puro) (Figure 1B). CYFP-tagged prey retroviruses were produced as mentioned above and used to infect the stable NYFP tagged ROP18_I_ (or ROP18_II_) bait cells. Two days after infection, the infected cells were subjected to 5–10 days of selection with 1 μg/mL puromycin to obtain the stable cell lines co-expressing NYFP-tagged ROP18_I_ (or ROP18_II_) and CYFP-tagged prey. All procedures were performed in 96-well plates, using the Biomek 3000 Laboratory Automation Workstation (Beckman Coulter, Brea, CA, USA). The resulting diploid cells were then harvested, and the fluorescent cells were sorted out using the LSRII flow cytometer equipped with a high-throughput sampler (BD Biosciences, San Jose, CA, USA), along with the HTC75 cells infected with only CYFP-EV retroviruses as the negative control group. The positive fluorescent cells were harvested and subjected to another round of sorting until the desired positive rate (more than 90%) was reached (Figure S1 in Supplementary Material). mRNAs of the final positive cells were extracted and reverse-transcribed into cDNA by RT-PCR amplification and were then identified through Illumina/Solexa sequencing (29).

To determine the false-positive BiFC signals resulting from the self-assembly of the two YFP fragments, a control screening was performed, in which a stable bait cell line was generated to express NYFP without fusion to ROP18_I_ or ROP18_II_. The expressed NYFP was then mated with each CYFP-prey/prey-CYFP in the prey library (Figure 1C). The Original Total Reads of each prey was calculated through the high-throughput sequencing analysis of the whole prey library, and the NYFP Total Reads were calculated through the sequencing analysis of the positive cells obtained from the control screening. A Bias Ratio was then defined as the tendency of the NYFP fragment to associate with the CYFP-prey/prey-CYFP, by comparing the NYFP Total Reads to the Original Total Reads for each prey. The higher the Bias Ratio, the higher risk of identifying the prey as a positive signal. The preys with a Bias Ratio of more than 1% were regarded as false-positives and discarded.

### SE-FRET Assay

The day before transfection, a total of 1 × 10^5^ COS-7 cells were seeded in each well of a 12-well plate with 1 mL DMEM growth medium (no antibiotics). When the cells were about 60% to 80% confluent, 1 μg of pEYFPC1-NMI (pEYFPC1-IL20RB, pEYFPC1-P2RX1, pEYFPC1-IL21, pEYFPC1-UBC, or pEYFPC1-vimentin) and/or 1 μg of pECFPN1-ROP18_I_ plasmids were transfected into COS-7 cells for the experimental groups, using Lipofectamine 2000 transfection reagent (#11668-019, Invitrogen, Waltham, MA, USA). For the negative control group, pECFPN1 and pEYFPC1 empty vectors were transfected into the cells, while for the positive control group, pECFPN1-EYFP was transfected into the cells. At 6 h post-transfection, the medium was replaced with fresh complete growth medium.

For the SE-FRET assay, prior to the testing of co-transfection samples, the images of the donor (CFP-ROP18_I_ only) and acceptor (YFP-prey only) channels were collected to determine the spectral bleed-through. The images of the donor, acceptor, and FRET channels were simultaneously collected for selection of the region of interest during detection of the samples co-transfected with CFP-ROP18_I_ and YFP-prey. The adjusted fluorescence density was obtained by subtraction of the background light density from the fluorescence density of the protein signal. The fluorescence signal, FRET efficiency, and distance between donor and acceptor were analyzed and calculated using the Olympus FluoView FV1000 viewer software (Olympus, Tokyo, Japan).

### Co-immunoprecipitation (Co-IP) Assay

COS-7 cells overexpressing ROP18_I_ and/or NMI (IL20RB, P2RX1, IL21, or vimentin) were prepared as mentioned previously in the FRET assay. Cell extracts were prepared by lysing the cells in cell lysis buffer (#P0013, Beyotime, Shanghai, China) with 1 mM phenylmethanesulfonyl fluoride (#WB-0181, Beijing Dingguo Changsheng Biotechnology, Beijing, China). Cell lysates were incubated with the primary antibody (anti-NMI rabbit monoclonal antibody anti-HA rabbit monoclonal antibody, anti-P2RX1 goat polyclonal antibody, or anti-FLAG mouse monoclonal antibody) with gentle rotation for 1 h at 4°C. Protein A-Agarose (#sc-2001, Santa Cruz Biotechnology, Santa Cruz, CA, USA) was then added to the immunoprecipitation reaction with incubation overnight at 4°C. The immunoprecipitates were washed four times with phosphate-buffered saline and then eluted by boiling with SDS-PAGE loading buffer (#9173, TAKARA, Kusatsu, Japan). The eluates were analyzed by western blotting with the indicated antibodies, as described previously (28). For the UBC experiment, cells were treated with 10 μM proteasome inhibitor MG132 (#S1748, Beyotime, Shanghai, China) for 12 h before harvesting.

### Data Analysis

Each protein sequence and functional information was obtained from the UniProt Database (http://www.uniprot.org/). To further define the biological functions of the *Tg*ROP18 interactome, the *Tg*ROP18-interacting proteins were analyzed using DAVID Bioinformatics Resources 6.8 (30, 31) for gene ontology (GO) annotation and enrichment analysis. Pathway analyses were done using Ingenuity Pathway Analysis (IPA, Ingenuity^®^ Systems, www.ingenuity.com) by importing the Entrez GeneID of the *Tg*ROP18-interacting proteins into online servers. Additionally, a combination of the search tool for the retrieval of interacting genes/proteins (STRING) version 10.0 database (32) and Cytoscape version 3.4.0 (33) was used to explore and build the PPI network. Statistical analysis data are presented as mean ± SD. Student’s *t-*test was utilized for statistical analysis to evaluate the significant difference between different groups using IBM SPSS Statistics 20.0 (34). Statistical significance was accepted if *p* < 0.05.

## Results

### Characterization of the *Tg*ROP18 Interactome

After multiple rounds of flow cytometric sorting, the final positive sorting rate of the HTC75 cells co-expressing ROP18_I_-NYFP and CYFP-tagged prey and the HTC75 cells co-expressing ROP18_II_-NYFP and CYFP-tagged prey were 93.2 and 98.6%, respectively (Figure S1 in Supplementary Material). After background noises in sequencing were filtered out using a cutoff value of five in total reads, 492 ROP18_I_ (2.88%) and 141 ROP18_II_ (0.83%) interacting proteins were identified, compared to control cells (Figure 1D). Tables S1 and S2 in Supplementary Material present the list of ROP18_I_- and ROP18_II_-interacting proteins with their total reads, respectively. Based on the specificity of the interaction, we classified the interacting proteins into three groups: A. 470 ROP18_I_-specific targets; B. 119 ROP18_II_-specific targets; and C. 22 targets for both ROP18_I_ and ROP18_II_ (Figure 1D). Regarding the ROP18_I_ specific targets, many were ribosomal proteins (e.g., RPL23, RPL11, RPL37A, RPS14, and RPS6), GTPases (e.g., SAR1B, ARL17B, and RND2), and receptors (e.g., PTPRF, FPR1, IL9R, IL20RB, and KLRD1), whereas for the ROP18_II_ specific targets, many were transmembrane proteins (e.g., TM4SF20, CMTM3, TMEM147, and TMBIM4) and zinc finger proteins (e.g., ZNF232, ZSCAN2, ZSCAN32, and ZNF273). Interestingly, we found that some humoral regulating factors (e.g., UTS2, CST2, and DEFB129) and some enzymes (e.g., DEGS1 and TPO) were targeted by both ROP18_I_ and ROP18_II_.

Among the ROP18_I_-interacting proteins, CNBP, DCTD, NUP160, and PRAC, which had been previously confirmed as ROP18_I_-interacting proteins by a previous human proteome array (22), were also identified in our HT-BiFC assay, indicating the reliability and quality of our results. In addition to these known interactions, 488 interactions of ROP18_I_ with human proteins were newly defined in our study. Notably, to our knowledge, our findings provided the first report of ROP18_II_-interacting proteins in human cells and specifically identified 141 ROP18_II_-interacting human proteins.

### Validation of the *Tg*ROP18-Interacting Proteins

To further validate the interactions identified by the HT-BiFC assay, six ROP18_I_-interacting proteins (NMI, IL20RB, P2RX1, IL21, UBC, and vimentin) with a broad range of total reads (169793, 30861, 25144, 2214, 116, and 6, respectively) were selected for two independent assays, the SE-FRET assay and the Co-IP assay.

**Figure 2:**
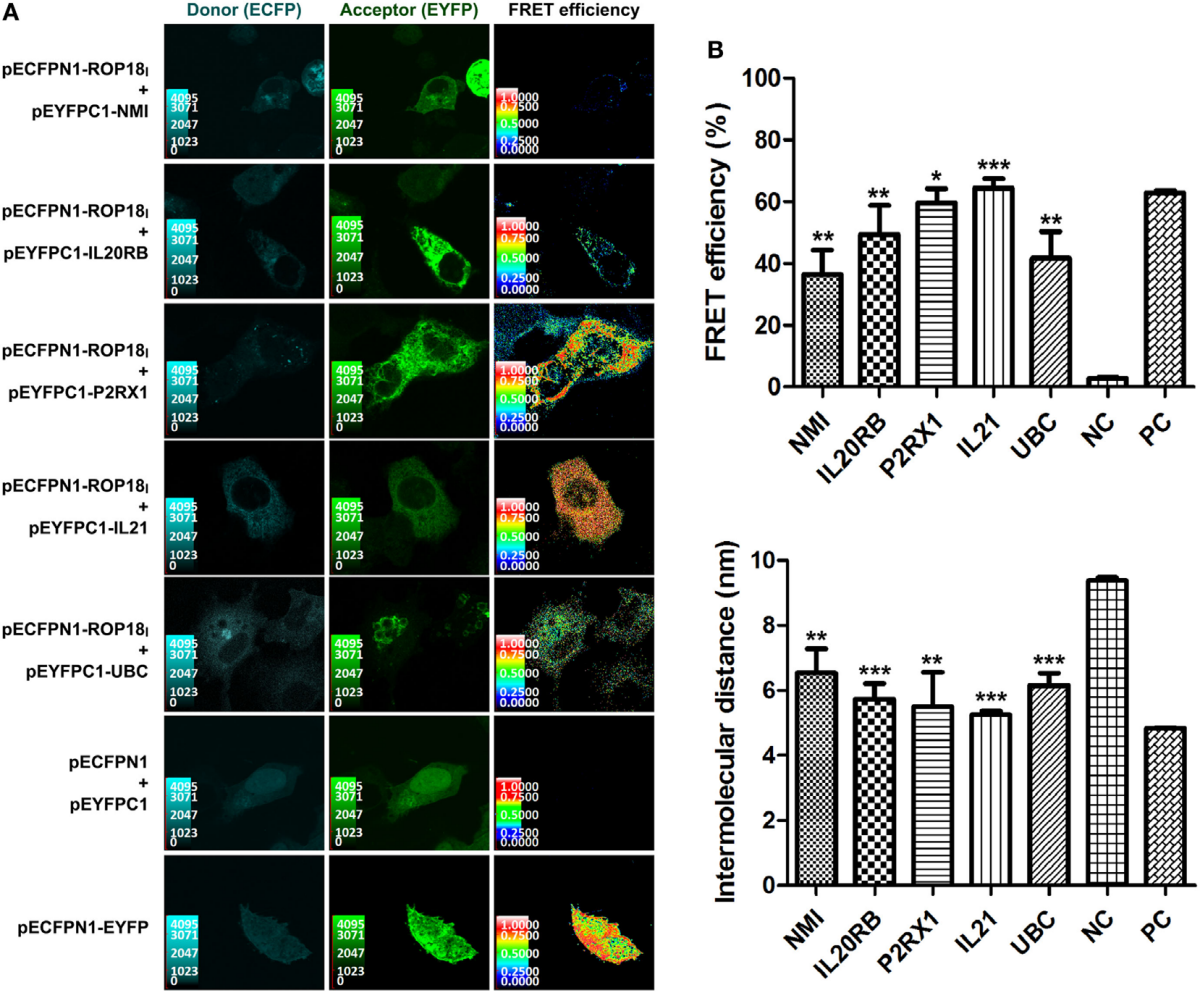
Validation of the *Tg*ROP18-interacting proteins by SE-FRET assay. **(A)** Co-localization and FRET interaction of ROP18_I_ with NMI, IL20RB, P2RX1, IL21, and UBC. Localization and co-localization of ROP18_I_ and the five indicated candidates are shown in the donor channel (column 1) and the acceptor channel (column 2), respectively. The FRET efficiency is shown in column 3, in which a thermal pseudo color-matched FRET signal intensity scale is indicated for each image. **(B)** Quantitative analysis of FRET efficiency and intermolecular distance between ROP18_I_ and the five indicated candidates. Error bars represent the means ± SD of triplicates. Student’s *t*-tests results are between the six experimental groups and NC, *p < 0.05; **p < 0.01; ***p < 0.001. Abbreviations: NC, negative control; PC, positive control.

In the SE-FRET assay (Figure 2A), the donor and acceptor channels show the co-localizations of ROP18_I_ with NMI, IL20RB, P2RX1, IL21, or UBC in the cytoplasm, suggesting the potential PPIs and their cytoplasmic localization. In addition, positive FRET signals were observed in the positive control cells and the COS-7 cells co-expressing ROP18_I_ and NMI, IL20RB, P2RX1, IL21, or UBC (Figure 2A), yielding significantly higher FRET efficiency and less intermolecular distance than negative control cells (*p* < 0.05, see Figure 2B). The SE-FRET results of ROP18_I_ and vimentin have been published recently in a study from our laboratory (35). These findings demonstrate the stable interactions of ROP18_I_ with NMI, IL20RB, P2RX1, IL21, UBC, and vimentin in the cytoplasm and are consistent with the HT-BiFC results, despite slight differences between the FRET efficiency values and total reads.

**Figure 3:**
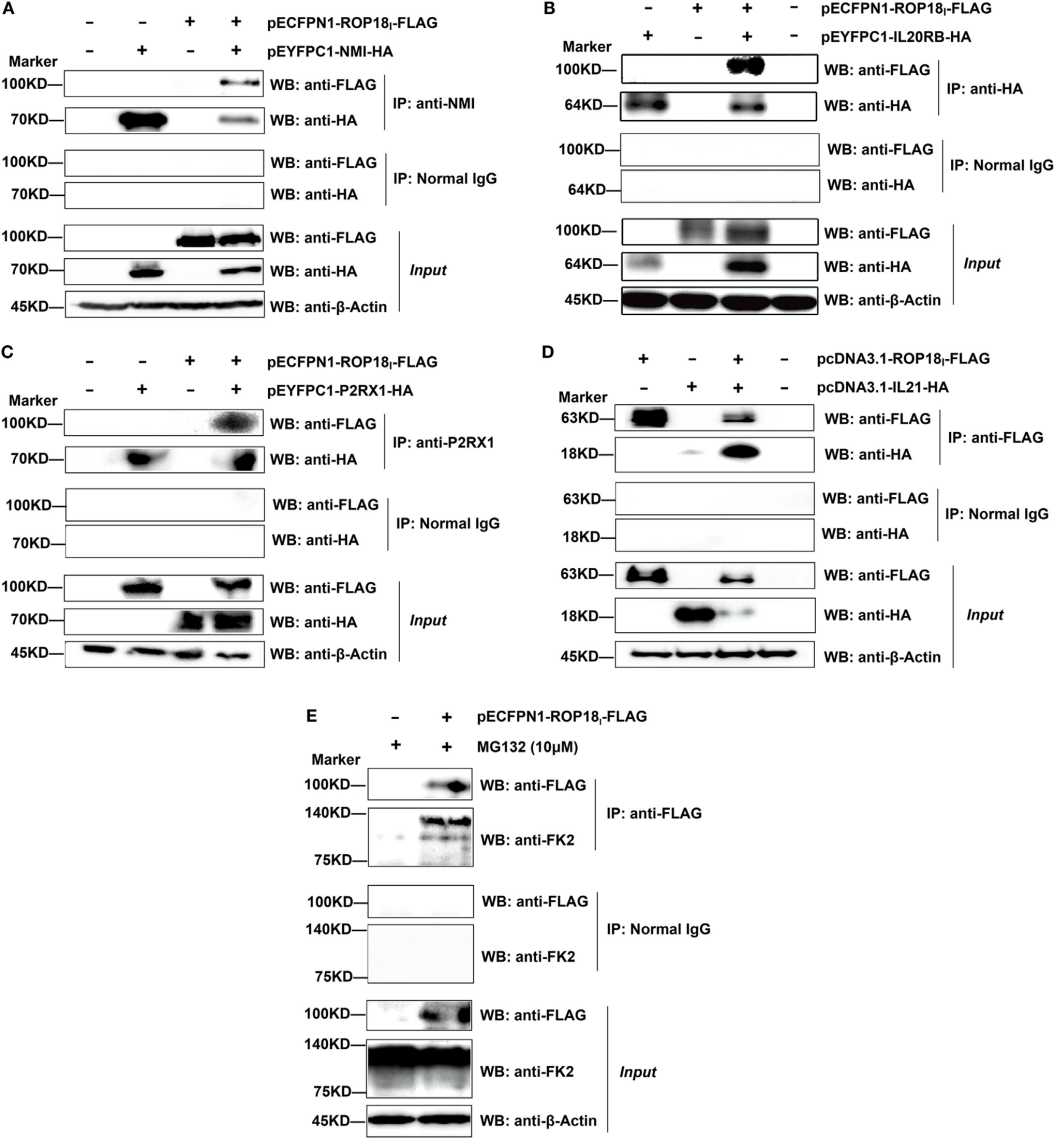
Validation of the *Tg*ROP18-interacting proteins by co-immunoprecipitation (Co-IP) assay. **(A–D)** Lysates of COS-7 cells co-overexpressing ROP18_I_ and the indicated interacting proteins were immunoprecipitated with the indicated antibodies. Rabbit, mouse, or goat normal control IgG were used as negative controls. The immunoprecipitates were detected by SDS-PAGE and western blotting using the antibodies indicated. **(E)** Lysates of COS-7 cells overexpressing ROP18_I_ in the presence of MG132 (10 μM) for 12 h were immunoprecipitated with the anti-FLAG antibody. Endogenous UBC (a smear of bands) was detected in the immunoprecipitates through western blotting with anti-FK2 antibody, which recognizes mono- and polyubiquitinylated conjugates.

The interactions were also confirmed by our three replicates of Co-IP assays (Figure 3). The results show that in the dually transfected cells, ROP18_I_ could be readily detected in the immunoprecipitates by using the specific antibodies anti-NMI, HA, P2RX1, and FLAG, but not with the control IgG. The Co-IP result of ROP18_I_ and vimentin has been published recently in a study from our laboratory (35). These results confirmed the consistency between the two assays, suggesting the robustness and reliability of the HT-BiFC results.

### Bioinformatic Analysis of the *Tg*ROP18-Interacting Proteins

**Figure 4:**
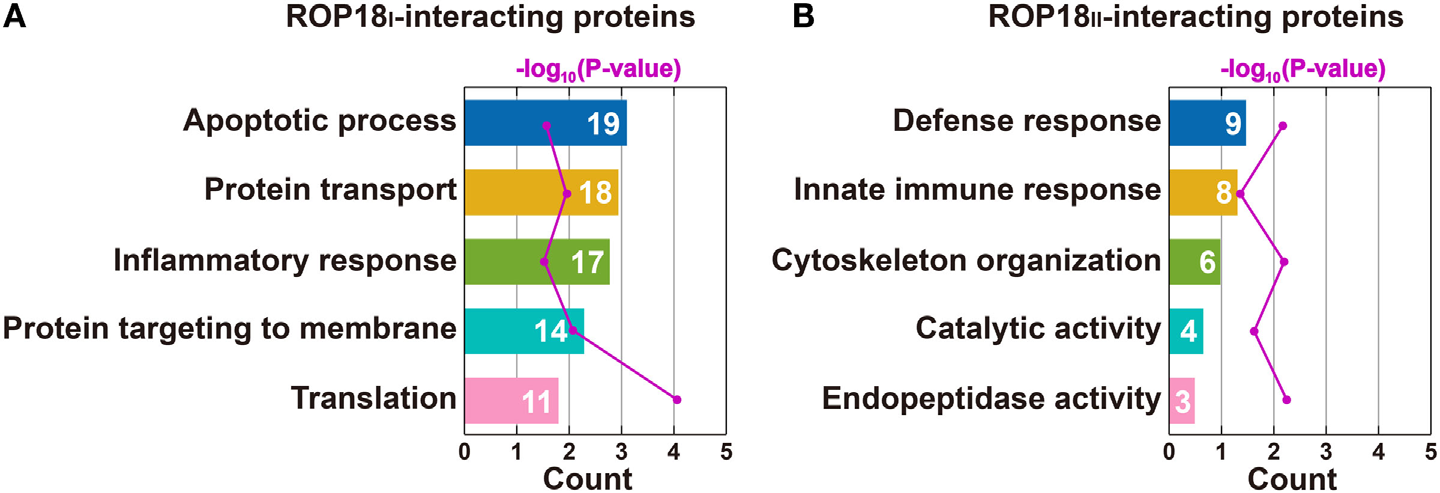
Top five enriched biological processes for ROP18_I_ **(A)** and ROP18_II_-interacting proteins **(B)** identified by GO analysis. **(A)** ROP18_I_-interacting proteins were significantly overrepresented in the biological processes of apoptotic process, protein transport, inflammatory response, protein targeting to membrane, and translation. **(B)** ROP18_II_-interacting proteins were significantly overrepresented in the biological processes of defense response, innate immune response, cytoskeleton organization, catalytic activity, and endopeptidase activity.

To obtain a comprehensive view of the *Tg*ROP18 interactome, we performed a GO analysis to identify significantly enriched functional terms of *Tg*ROP18-interacting proteins. The top five enriched terms within the “Biological Process” ontology category, together with their protein counts and *p*-values, are shown in Figure 4. The results reveal that both ROP18_I_ and ROP18_II_-interacting proteins were significantly enriched in a variety of biological processes (*p* < 0.05). As expected, ROP18_I_-interacting proteins were significantly overrepresented in the biological processes of apoptotic process (*p* = 2.7 × 10^−2^), inflammatory response (*p* = 1.1 × 10^−2^), and protein targeting to membrane (*p* = 8.7 × 10^−5^), and for ROP18_II_, we also added host targets to the expected biological processes, including defense response (*p* = 7.8 × 10^−3^) and innate immune response (*p* = 4.0 × 10^−2^). In addition to the roles of *Tg*ROP18 in the expected biological processes mentioned above, interesting roles of ROP18_I_ in protein transport (*p* = 3.0 × 10^−2^) and translation (*p* = 8.5 × 10^−5^), and ROP18_II_ in cytoskeleton organization (*p* = 6.9 × 10^−3^), catalytic activity (*p* = 2.2 × 10^−2^), and endopeptidase activity (*p* = 6.1 × 10^−3^) were also identified with great significance.

**Figure 5:**
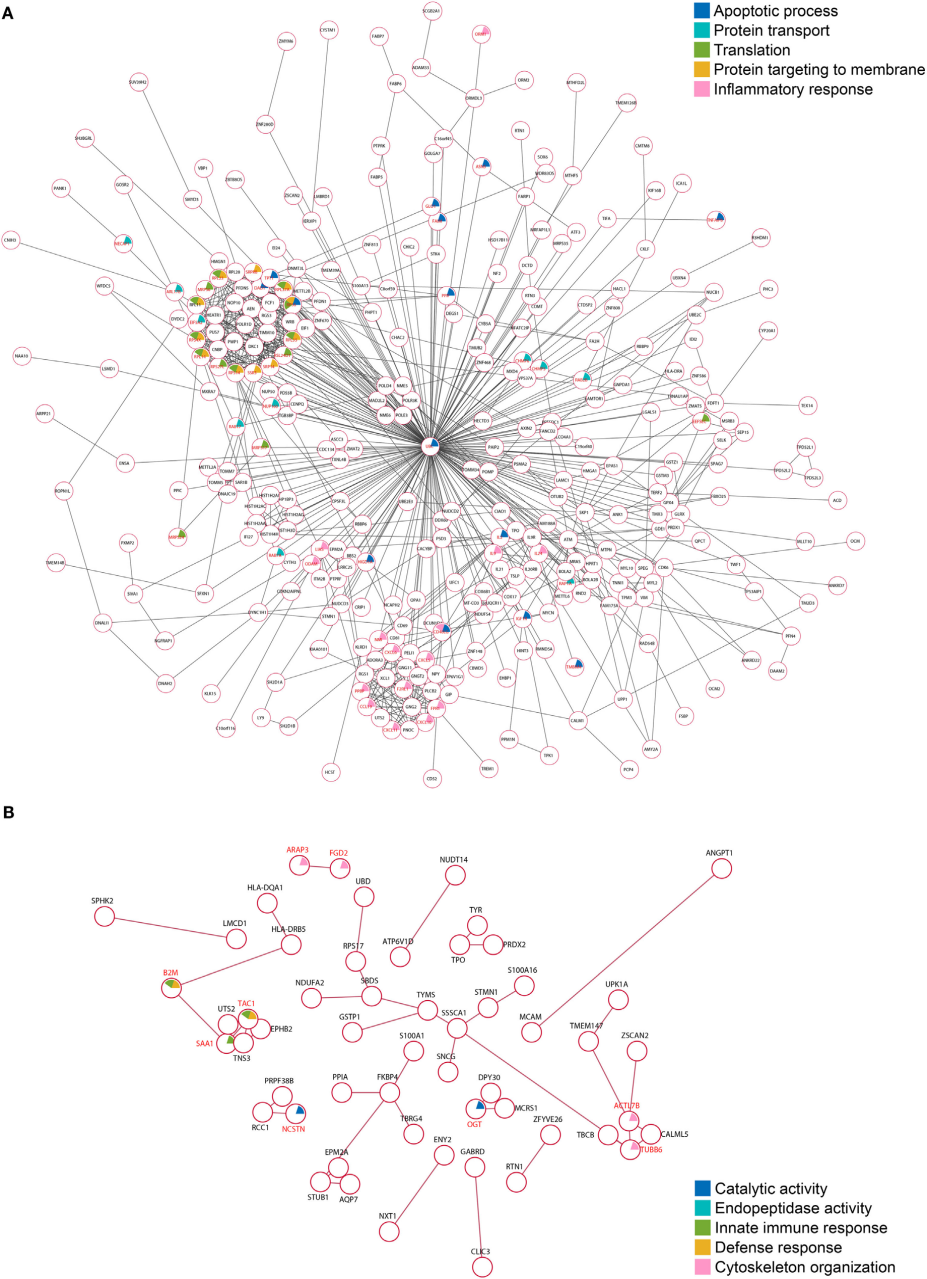
Protein–protein interaction (PPI) networks of the *Tg*ROP18-interacting proteins. **(A)** Among the ROP18_I_-interacting proteins, 353 (71.7%) are tied to a single large network with 785 edges. **(B)** Among the ROP18_II_-interacting proteins, 55 (39.0%) are enriched in a PPI network with 44 edges.

To elucidate whether the *Tg*ROP18-interacting proteins were functionally related, we conducted a deeper exploration of the PPI networks by using the STRING 10.0 database. By applying a medium confidence (*p* > 0.4), 353 (71.7%) of the ROP18_I_-interacting proteins were tied to a single large network with a PPI enrichment *p*-value < 0.001; whereas for the ROP18_II_-interacting proteins, 55 (39.0%) were enriched in a large PPI network, with a PPI enrichment *p*-value of 0.009 (Figure 5), which indicated that each one of the two sets of interacting proteins were biologically connected as a network with a significantly greater number of interactions, rather than as a random set of proteins. As shown in Figure 5A, 785 edges (PPIs) were observed among the 353 ROP18_I_-interacting proteins, and seven protein–protein-interacting clusters were evident in the network, such as a cluster of ribosomal proteins containing RPS4X, RPL35, RPL23, RPL37A, and RPS6; a cluster of chemokines containing CXCL6, CXCL5, CCL19, CXCL11, and CXCL10; and a cluster of interleukins containing IL2, IL9, IL21, and IL24. Notably, UBC was observed as a main hub situated in the core of the network with 211 edges. Among the 55 ROP18_II_-interacting proteins, 44 edges and four protein–protein-interacting clusters were determined by STRING analysis (Figure 5B). The cytoskeleton proteins ACTL7B, TUBB6, and TBCB were in close proximity and formed a cluster; TNS3 and UTS2 tensins were closely clustered with a tachykinin, TAC1; several functional regulators, such as TBRG4, PPIA, S100A1, and FKBP4 were tied together as a cluster; and several disease-related proteins, such as SNCG, STMN1, SSSCA1, and S100A16, were identified to be clustered.

**Figure 6:**
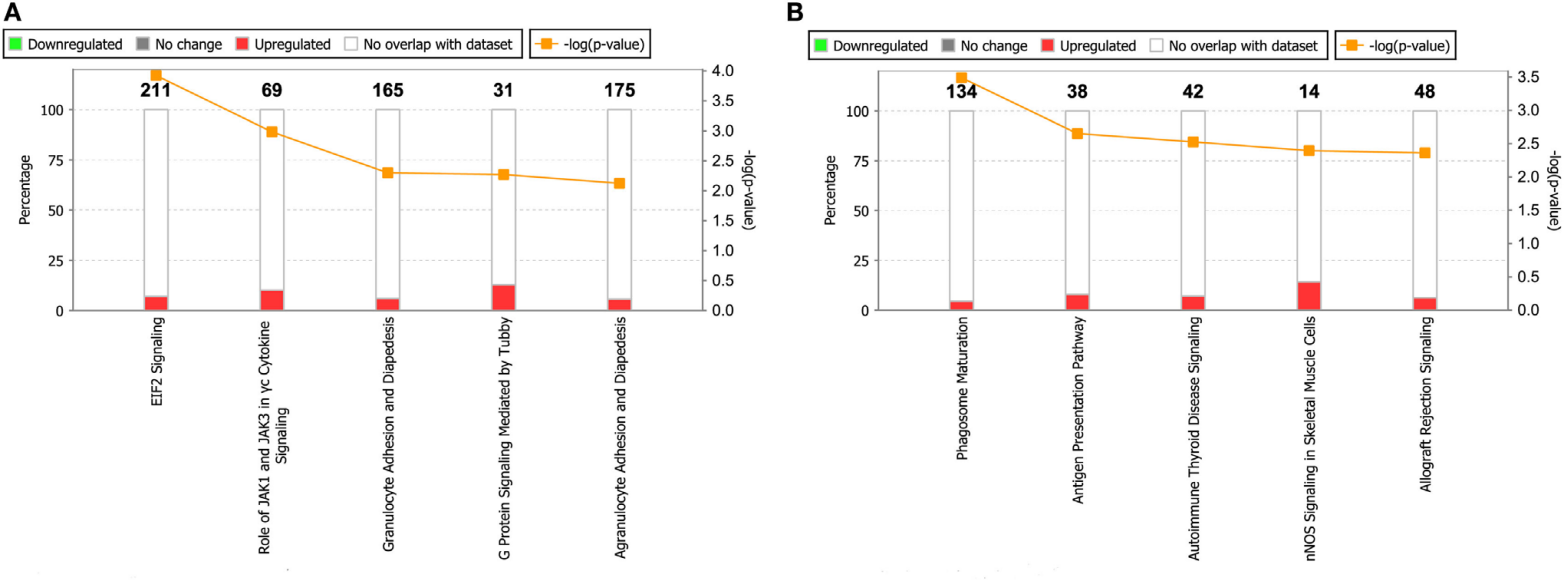
Top five enriched pathways for ROP18_I_ **(A)** and ROP18_II_ **(B)** interacting proteins. The stacked bar chart indicates the number of proteins overlapped with the database, and the connected orange points represent the logarithm of the *p*-values.

By using the IPA database, we carried out an Ingenuity pathway analysis to further investigate the significant human signaling pathways influenced by ROP18_I_/ROP18_II_. Among the 492 ROP18_I_-interacting proteins, 71 (14.4%) were mapped to 34 pathways, and 13 (9.2%) out of the 141 ROP18_II_-interacting proteins were mapped to 16 pathways in total. All the involved significant pathways with their *p*-values and associated molecules are listed in Table S3 in Supplementary Material. The top five enriched pathways for the ROP18_I_ and ROP18_II_-interacting proteins shown in Figure 6 were closely related to cell growth, cytokine signaling, and cellular immune response. For the ROP18_I_-interacting proteins, MRAS was involved in the higher number of pathways, followed by ATM; whereas for the ROP18_II_-interacting proteins, human leukocyte antigen (HLA)-DRB5 was involved in the higher number of pathways, followed by HLA-DQA1.

## Discussion

Protein–protein interaction plays indispensable roles in structuring and regulating biological processes in all biological systems. The “protein-protein interactome” refers to the whole union of all PPIs in a particular cell or organism (36). In addition to serving as a foundation for more detailed studies on the prediction of protein functions or disease associated genes (37, 38), interactome mapping has become a critical and powerful postgenomic research tool that facilitates a better understanding of genotype-to-phenotype relationship and biological systems (39). *Tg*ROP18, which is a key virulence determinant of *T. gondii*, modulates the host cell and mediates the parasite virulence by interacting with host proteins. However, only a few host targets of ROP18_I_ have been identified, and knowledge about the host targets of ROP18_II_ is still very limited.

The BiFC technique is an effective and robust tool for studying PPIs, as it enables not only the direct visualization of the occurrence and subcellular localization of PPIs in live cells (40, 41) but also the detection of weak or transient interactions due to the strong signal and high stability of the reconstituted fluorescent complex (25, 42). In the present study, we used a genome-wide BiFC-based proteomic approach to profile the *Tg*ROP18 (ROP18_I_ and ROP18_II_) interactome in human cells. Compared with control cells, a total of 492 ROP18_I_ and 141 ROP18_II_-interacting proteins were identified. Among these proteins, six of them, NMI, IL20RB, P2RX1, IL21, UBC, and vimentin, were further confirmed as authentic ROP18_I_ targets by our SE-FRET and Co-IP assays and, furthermore, four of them, CNBP, DCTD, NUP160, and PRAC, had been previously reported as *Tg*ROP18 substrates by a human protein array (22). All of these results confirming the *Tg*ROP18 targets have strongly supported the power of our HT-BiFC assay. In this HT-BiFC assay, the NYFP- or CYFP-tagged prey collections covered 93% of all human ORFs, facilitating a much more complete and previously unavailable description of the *Tg*ROP18 interactome. We also newly identified 488 ROP18_I_-interacting proteins when compared to the two previous screenings for ROP18_I_ targets (20, 22). Such findings have demonstrated the significant advantages of the BiFC system to detect not only strong binding, but also transient or weak PPIs that would often be missed by using YTH and protein arrays (20, 22). The discovery of the novel *Tg*ROP18-interacting proteins have also shown the differences between the experimental methods used in the present study and in previous studies using YTH (20) and protein array methods (22), which analyzed the PPIs in yeast or *in vitro*. To our knowledge, ours is the first study to report the *Tg*ROP18 interactome in the natural cellular context. Our data appears to be highly complementary to the existing information about *Tg*ROP18, and the newly identified *Tg*ROP18-interacting proteins will be potential candidates for further investigations into the regulatory roles of *Tg*ROP18 in human cells.

### *Tg*ROP18 and Immune Response

**Figure 7:**
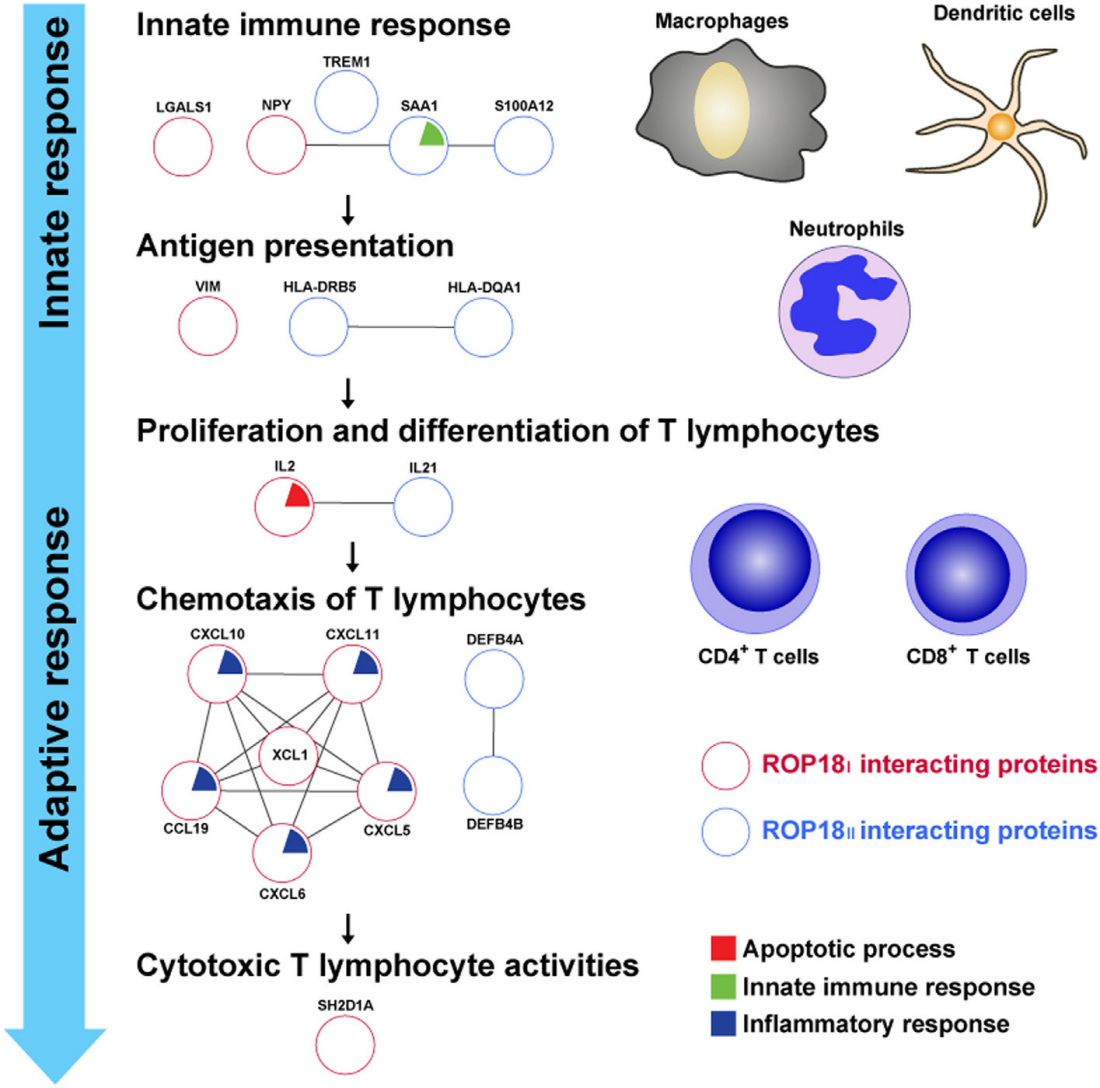
*Tg*ROP18-interacting proteins involved in the immune response. Numerous ROP18_I_ (red circles) and ROP18_II_ (blue circles) identified interacting proteins related to the processes of immune response. During *T. gondii* infection, innate immune response acts rapidly to provide the first line of defense and activate the adaptive immune response, with release of proinflammatory cytokines by macrophages, dendritic cells, and neutrophils. In the milieu of proinflammatory cytokines, T cell-mediated immune response is initiated when naïve CD4^+^ or CD8^+^ T cells encounter the parasite antigens presented by antigen presenting cells (APCs). Once the antigen-specific T lymphocytes are activated, they proliferate, differentiate, and traffic to the sites of infection, protecting the host by exhibiting cytotoxic T lymphocyte (CTL) activities toward infected cells.

During *T. gondii* infection, immune defense against the parasite is strongly induced in its mammalian hosts, and T cell-mediated immune response plays a key role in this defense (43, 44). In our results, most *Tg*ROP18-interacting proteins were associated to the processes of immune response, including innate immune response, antigen presentation, activation and chemotaxis of naïve T lymphocytes, and cytotoxic reaction of effector T lymphocytes (Figure 7).

Innate immune response provides a first line of defense against *T. gondii* infection and is essential for the activation of the adaptive immune response (45). Following the infection of *T. gondii*, innate cells, including macrophages, dendritic cells, and neutrophils, are recruited to the sites of infection, producing proinflammatory cytokines, phagocytizing the parasites, or generating reactive chemical substances in order to inhibit the replication and dissemination of the parasites (46–49). Human NPY, LGALS1, S100A12, SAA1, and TREM1 have been reported as regulators of innate immunity, modulating the innate immune functions by controlling the innate cells physiology and cytokines release (50–55). In this study, NPY, LGALS1, and TREM1 were found as targets of ROP18_I_, and S100A12, SAA1, and TREM1 were targets of ROP18_II_. These results indicate a potential role of *Tg*ROP18 in manipulating and disarming the host innate immune response, which may contribute to the increase in parasites’ survival in infected cells.

Antigen presentation is the first step for induction of T cell-mediated response (56). Infection with *T. gondii* provides a strong stimulus for antigen-specific CD4^+^ and CD8^+^ T cells, which suggests that the parasite antigens are efficiently acquired by APCs and presented to antigen-specific T lymphocytes during infection. It has been reported that intermediate filament protein vimentin plays a key role in antigen presentation, and disruption of vimentin in Langerhans cells results in failed antigen presentation of these cells (57). HLA molecules have also been known to carry out an indispensable role in antigen processing and presentation, by binding the pathogen antigens and displaying them on the cell surface for recognition by T lymphocytes (56). In this study, we found an interaction between ROP18_I_ and vimentin, and this interaction was confirmed by FRET and Co-IP assays (35). Moreover, HLA-DQA1 and HLA-DRB5 were identified as the targets of ROP18_II_. These results suggest that *Tg*ROP18 may confer the virulence to the parasite and exert its influence on human cellular immunity by forming PPIs with the key proteins involved in antigen processing and presentation.

Once the T lymphocytes are specifically sensitized by exposure to the parasite antigens, they undergo proliferation and differentiation, which is regulated by inflammatory cytokines (58). Proinflammatory cytokines, such as IL-2 and IL-21, are pivotal mediators in triggering development of T cell populations and effector functions against *T. gondii* infection mediated by T lymphocytes. Though the IL-2 response is not potently induced during *T. gondii* infection (59), IL-2^−/−^ mice have a defect in production of IFN-γ and exhibit poor CD8^+^ T cell responses against the parasite (60). In addition, Khan et al. have reported that in mice lacking functional IL-21, expression of co-stimulatory molecules on CD8^+^ T cells is strongly downregulated by *T. gondii* infection, and in the absence of IL-21 receptors, the functions of CD8^+^ T cells are significantly affected (61). In the present study, we identified IL-2 and IL-21 as the host targets of ROP18_I_. In particular, IL-21 was further confirmed as the ROP18_I_-interacting protein by FRET and Co-IP assays (Figures 2 and 3). These data suggest that ROP18_I_ may inhibit the activation and development of host T lymphocytes by targeting the key cytokines, resulting in the dysregulation of T cell-mediated immunity.

During *T. gondii* infection, multiple chemokines are upregulated, contributing to T cells entry into the sites of infection and targeting of parasites (62). In murine ocular and cerebral toxoplasmosis, there is a significant increase in the expression levels of CXCL10 and CXCL11 over the course of infection (63, 64). CCL19 is a vital chemokine in multiple immunological processes, including generation of thymocytes, promotion of regulatory T cells activity, and homing of leukocytes (65–67). The family of β-defensins (DEFB) consists of a number of cationic host defense peptides, such as DEFB4A and DEFB4B, which play a dual role in both innate and adaptive immune response (68). In this study, chemokines CXCL5, CXCL6, CXCL10, CXCL11, CCL19, and XCL1 were identified as the ROP18_I_-interacting proteins, and DEFB4A and DEFB4B were identified as the ROP18_II_ targets, which indicate a regulatory role of *Tg*ROP18 in human chemokines, enabling the parasite to interfere with the host immune responses and finally promote parasite’s survival.

After being attracted to the sites of infection, the antigen-specific effector T lymphocytes display strong effector functions toward infected cells for host protection (69). SH2D1A (or signaling lymphocytic activation molecule-associated protein, SAP) is an adaptor protein that regulates signaling through signaling lymphocytic activation molecule family receptors expressed on T lymphocytes and NK cells (70). Mutations in the *sh2d1a* gene or lack of SH1D2A protein show a significant decrease in the production of IFN-γ, resulting in disruption of cytotoxic T lymphocyte (CTL) function and defective lytic activity against EBV-positive target cells (71). In our study, we identified SH2D1A as a host target of ROP18_I_. Given the importance of SH2D1A in CTL activities, being targeted by ROP18_I_ may lead to impaired function of SH2D1A, thereby decreasing cytotoxic activity against *T. gondii* infection.

### *Tg*ROP18 and Apoptosis

Apoptosis is a programmed, regulated form of cell death that permits the active and safe self-destruction of the cell (72). It plays a major role in cell development, tissue homeostasis, immune defense, and protection against tumorigenesis (73). *T. gondii* appears to use various strategies to interfere with host cell apoptosis through both pro-apoptotic and anti-apoptotic activities. Such complex dual activities of the parasite may be crucial for stable host-parasite interaction and sustained toxoplasmosis (74, 75). After acute infection, increased apoptosis of immune cells induced by *T. gondii* may suppress the immune responses against the parasite, thereby leading to immune evasion. On the other hand, inhibition of host cell apoptosis may serve as a mechanism for preserving intracellular replication and long-term survival of the parasite (76).

*Tg*ROP18 has been shown to use a variety of mechanisms, including the mitochondrial pathway, to modulate the host cell apoptosis (16). As a mitochondrial inner membrane protein, HIGD1A inhibits cytochrome c release and reduces caspases activities, thus suppressing the mitochondrial pathway of apoptosis (77). IGF1R, which is a tyrosine kinase, acts as an anti-apoptotic agent by upregulating the expression of anti-apoptotic members of the BCL2 family. Inhibition of IGF1R not just leads to reduced anti-apoptotic BCL2 proteins, but also increases expression of proapoptotic Bax/Bak-like BCL2 proteins and cleavage of caspase 3 (78). In our research, we found that HIGD1A and IGFIR were targeted by ROP18_I_, suggesting that these host proteins might be significant for ROP18_I_ to manipulate human cell apoptosis through the mitochondrial pathway.

The death receptor pathway is another major pathway of apoptosis. Fas apoptotic inhibitory molecule (FAIM) is a death receptor antagonist that protects the cell from Fas-induced apoptosis by inhibiting auto-ubiquitinylation and proteasome-dependent degradation of the apoptotic suppressor protein XIAP (79). Cells overexpressing FAIM show increased resistance to apoptosis triggered by the death receptor, and suppression of FAIM expression protects the cell against death receptor-induced apoptotic cell death (80). We found an interaction between ROP18_I_ and FAIM in this study, which suggested that the parasite might interfere with the death receptor pathway of host cell apoptosis through targeting the key component in this pathway by *Tg*ROP18.

The ER is a central cellular organelle responsible for several crucial biological processes, and ER stress condition can trigger cell apoptosis when the stress is prolonged and severe (81). It has been reported that *T. gondii* can induce apoptosis of host cells *via* the ER stress pathway by upregulating the expression of C/EBP homologous protein, c-JUN NH2-terminal kinase, and activated caspase 12 (82, 83). Furthermore, ROP18_I_ exerts a facilitated effect on the ER stress-induced apoptosis of the host cell by increasing the expression levels of the key molecules involved in the pathway (84). Consistent with this, two human proteins, PPT1 and PSMD10, which were involved in the ER stress-induced apoptosis, were identified as the ROP18_II_-interacting proteins in our HT-BiFC assay. PPT1 is a lysosomal enzyme that is associated with the depalmitoylation and degradation of S-acylated proteins. PSMD10, also named as p28, suppresses ER stress-induced apoptosis by upregulating the expression of GRP78 and promoting the recovery of the cell from ER stress (85). Considering the significant roles of PPT1 and PSMD10 in ER stress-induced apoptosis, disruption of PPT1 and PSMD10 by ROP18_II_ may facilitate the ER stress-induced apoptosis of host cells, leading to restricted immune responses and high parasite burden. Our study suggests a pleiotropic role of *Tg*ROP18 in altering the host cell apoptosis through multiple targets and pathways, providing a new and better understanding of this pathological process.

## Conclusion

Identification of the host targets of *T. gondii* effectors is important to reveal host-parasite interaction. We used high-throughput PPI screening based on BiFC for the first time to identify the human host proteins targeted by the *T. gondii* key virulence factor *Tg*ROP18. In total, 492 and 141 human proteins were identified as the targets of ROP18_I_ and ROP18_II_, respectively. These *Tg*ROP18-interacting proteins were involved in crucial pathways related to immune response and apoptosis. Our findings characterized an interactome of *Tg*ROP18 in human cells and described novel regulatory roles of *Tg*ROP18 on host cell functions. The analysis of the ROP18_I_ and ROP18_II_ PPIs networks would be useful to reveal the strategies of *T. gondii* virulence elicitation and the regulatory mechanisms of human responses to *T. gondii* infection.

## Ethics Statement

This article does not contain any experiments with human participants or animal subjects performed by any of the authors.

## Author Contributions

JX designed and performed experiments, analyzed data, and drafted manuscript. LK performed FRET and Co-IP assays of IL20RB, and graphed data. L-JZ performed FRET and Co-IP assays of P2RX1. S-ZW performed FRET and Co-IP assays of IL21. L-JY performed FRET assay of UBC. CH performed FRET and Co-IP assays of vimentin. CYH revised manuscript. H-JP designed experiments, revised manuscript, and submitted manuscript. All the authors read and approved the final version of the manuscript.

## Conflict of Interest Statement

The authors declare that the research was conducted in the absence of any commercial or financial relationships that could be construed as a potential conflict of interest.

## Supplementary Material

Figure S1 Flow cytometry histograms of HTC75 cells co-expressing ROP18_I_- (upper panel) or ROP18_II_-NYFP (lower panel) and Prey-CYFP, or expressing control constructs CYFP-EV, showing the ultimate positive sorting rate was more than 90%. EV, empty vector.

Table S1 List of ROP18_I_-interacting proteins identified by HT-BiFC assay.

Table S2 List of ROP18_II_-interacting proteins identified by HT-BiFC assay.

Table S3 List of significant pathways related to ROP18_I_ or ROP18_II_-interacting proteins.

## References

[1] Moncada PA Montoya JG. Toxoplasmosis in the fetus and newborn: an update on prevalence, diagnosis and treatment. Expert Rev Anti Infect Ther (2012) 10:815–28. 10.1586/eri.12.58 22943404

[2] Morrison DA. Evolution of the apicomplexa: where are we now? Trends Parasitol (2009) 25:375–82. 10.1016/j.pt.2009.05.010 19635681

[3] Nichols BA Chiappino ML O’Connor GR. Secretion from the rhoptries of *Toxoplasma gondii* during host-cell invasion. J Ultrastruct Res (1983) 83:85–98. 10.1016/S0022-5320(83)90067-9 6854716

[4] Boothroyd JC Dubremetz JF. Kiss and spit: the dual roles of *Toxoplasma* rhoptries. Nat Rev Microbiol (2008) 6:79–88. 10.1038/nrmicro1800 18059289

[5] Howe DK Sibley LD. *Toxoplasma gondii* comprises three clonal lineages: correlation of parasite genotype with human disease. J Infect Dis (1995) 172:1561–6. 10.1093/infdis/172.6.1561 7594717

[6] Barragan A Sibley LD. Migration of *Toxoplasma gondii* across biological barriers. Trends Microbiol (2003) 11:426–30. 10.1016/S0966-842X(03)00205-1 13678858

[7] Howe DK Summers BC Sibley LD. Acute virulence in mice is associated with markers on chromosome VIII in *Toxoplasma gondii*. Infect Immun (1996) 64:5193–8. 8945565 10.1128/iai.64.12.5193-5198.1996 PMC174507

[8] Mordue DG Monroy F La Regina M Dinarello CA Sibley LD Acute toxoplasmosis leads to lethal overproduction of Th1 cytokines. J Immunol (1950) 2001(167):4574–84. 10.4049/jimmunol.167.8.4574 11591786

[9] Sibley LD Ajioka JW. Population structure of *Toxoplasma gondii*: clonal expansion driven by infrequent recombination and selective sweeps. Annu Rev Microbiol (2008) 62:329–51. 10.1146/annurev.micro.62.081307.162925 18544039

[10] Sibley LD Boothroyd JC. Virulent strains of *Toxoplasma gondii* comprise a single clonal lineage. Nature (1992) 359:82–5. 10.1038/359082a0 1355855

[11] Saeij JP Boyle JP Coller S Taylor S Sibley LD Brooke-Powell ET Polymorphic secreted kinases are key virulence factors in toxoplasmosis. Science (2006) 314:1780–3. 10.1126/science.1133690 17170306 PMC2646183

[12] Taylor S Barragan A Su C Fux B Fentress SJ Tang K A secreted serine-threonine kinase determines virulence in the eukaryotic pathogen *Toxoplasma gondii*. Science (2006) 314:1776–80. 10.1126/science.1133643 17170305

[13] El Hajj H Lebrun M Arold ST Vial H Labesse G Dubremetz JF. ROP18 is a rhoptry kinase controlling the intracellular proliferation of *Toxoplasma gondii*. PLoS Pathog (2007) 3:e14. 10.1371/journal.ppat.0030014 17305424 PMC1797617

[14] Fentress SJ Behnke MS Dunay IR Mashayekhi M Rommereim LM Fox BA Phosphorylation of immunity-related GTPases by a *Toxoplasma gondii*-secreted kinase promotes macrophage survival and virulence. Cell Host Microbe (2010) 8:484–95. 10.1016/j.chom.2010.11.005 21147463 PMC3013631

[15] Steinfeldt T Konen-Waisman S Tong L Pawlowski N Lamkemeyer T Sibley LD Phosphorylation of mouse immunity-related GTPase (IRG) resistance proteins is an evasion strategy for virulent *Toxoplasma gondii*. PLoS Biol (2010) 8:e1000576. 10.1371/journal.pbio.1000576 21203588 PMC3006384

[16] Wu L Wang X Li Y Liu Y Su D Fu T *Toxoplasma gondii* ROP18: potential to manipulate host cell mitochondrial apoptosis. Parasitol Res (2016) 115:2415–22. 10.1007/s00436-016-4993-6 27021182

[17] Yamamoto M Ma JS Mueller C Kamiyama N Saiga H Kubo E ATF6beta is a host cellular target of the *Toxoplasma gondii* virulence factor ROP18. J Exp Med (2011) 208:1533–46. 10.1084/jem.20101660 21670204 PMC3135360

[18] Du J An R Chen L Shen Y Chen Y Cheng L *Toxoplasma gondii* virulence factor ROP18 inhibits the host NF-kappaB pathway by promoting p65 degradation. J Biol Chem (2014) 289:12578–92. 10.1074/jbc.M113.544718 24648522 PMC4007449

[19] Niedelman W Gold DA Rosowski EE Sprokholt JK Lim D Farid Arenas A The rhoptry proteins ROP18 and ROP5 mediate *Toxoplasma gondii* evasion of the murine, but not the human, interferon-gamma response. PLoS Pathog (2012) 8:e1002784. 10.1371/journal.ppat.1002784 22761577 PMC3386190

[20] Cheng L Chen Y Chen L Shen Y Shen J An R Interactions between the ROP18 kinase and host cell proteins that aid in the parasitism of *Toxoplasma gondii*. Acta Trop (2012) 122:255–60. 10.1016/j.actatropica.2012.02.065 22365922

[21] Fields S Song O. A novel genetic system to detect protein-protein interactions. Nature (1989) 340:245–6. 10.1038/340245a0 2547163

[22] Yang Z Hou Y Hao T Rho HS Wan J Luan Y A human proteome array approach to identifying key host proteins targeted by *Toxoplasma* kinase ROP18. Mol Cell Proteomics (2017) 16(3):469–84. 10.1074/mcp.M116.063602 28087594 PMC5341007

[23] Howell JM Winstone TL Coorssen JR Turner RJ. An evaluation of in vitro protein-protein interaction techniques: assessing contaminating background proteins. Proteomics (2006) 6:2050–69. 10.1002/pmic.200500517 16518870

[24] Kerppola TK. Design and implementation of bimolecular fluorescence complementation (BiFC) assays for the visualization of protein interactions in living cells. Nat Protoc (2006) 1:1278–86. 10.1038/nprot.2006.201 17406412 PMC2518326

[25] Kodama Y Hu CD. Bimolecular fluorescence complementation (BiFC): a 5-year update and future perspectives. Biotechniques (2012) 53:285–98. 10.2144/000113943 23148879

[26] Lee OH Kim H He Q Baek HJ Yang D Chen LY Genome-wide YFP fluorescence complementation screen identifies new regulators for telomere signaling in human cells. Mol Cell Proteomics (2011) 10:M110.001628. 10.1074/mcp.M110.001628 21044950 PMC3033672

[27] Yang X Boehm JS Yang X Salehi-Ashtiani K Hao T Shen Y A public genome-scale lentiviral expression library of human ORFs. Nat Methods (2011) 8:659–61. 10.1038/nmeth.1638 21706014 PMC3234135

[28] Na RH Zhu GH Luo JX Meng XJ Cui L Peng HJ Enzymatically active Rho and Rac small-GTPases are involved in the establishment of the vacuolar membrane after *Toxoplasma gondii* invasion of host cells. BMC Microbiol (2013) 13:125. 10.1186/1471-2180-13-125 23721065 PMC3681593

[29] Balasubramanian S Solexa sequencing: decoding genomes on a population scale. Clin Chem (2015) 61:21–4. 10.1373/clinchem.2014.221747 25332311

[30] Huang da W Sherman BT Lempicki RA. Bioinformatics enrichment tools: paths toward the comprehensive functional analysis of large gene lists. Nucleic Acids Res (2009) 37:1–13. 10.1093/nar/gkn923 19033363 PMC2615629

[31] Huang da W Sherman BT Lempicki RA. Systematic and integrative analysis of large gene lists using DAVID bioinformatics resources. Nat Protoc (2009) 4:44–57. 10.1038/nprot.2008.211 19131956

[32] Szklarczyk D Franceschini A Wyder S Forslund K Heller D Huerta-Cepas J STRING v10: protein-protein interaction networks, integrated over the tree of life. Nucleic Acids Res (2015) 43:D447–52. 10.1093/nar/gku1003 25352553 PMC4383874

[33] Shannon P Markiel A Ozier O Baliga NS Wang JT Ramage D Cytoscape: a software environment for integrated models of biomolecular interaction networks. Genome Res (2003) 13:2498–504. 10.1101/gr.1239303 14597658 PMC403769

[34] IBM SPSS Statistics for Windows. Version 20.0 ed. Armonk, New York: IBM Corporation (2011).

[35] He C Kong L Zhou L Xia J Wei H Liu M Host cell vimentin restrains *Toxoplasma gondii* invasion and phosphorylation of vimentin is partially regulated by interaction with TgROP18. Int J Biol Sci (2017) 13:1126–37. 10.7150/ijbs.21247 29104504 PMC5666328

[36] Vidal M Cusick ME Barabasi AL Interactome networks and human disease. Cell (2011) 144:986–98. 10.1016/j.cell.2011.02.016 21414488 PMC3102045

[37] Mostafavi S Ray D Warde-Farley D Grouios C Morris Q. GeneMANIA: a real-time multiple association network integration algorithm for predicting gene function. Genome Biol (2008) 9(Suppl 1):S4. 10.1186/gb-2008-9-s1-s4 18613948 PMC2447538

[38] Vanunu O Magger O Ruppin E Shlomi T Sharan R. Associating genes and protein complexes with disease via network propagation. PLoS Comput Biol (2010) 6:e1000641. 10.1371/journal.pcbi.1000641 20090828 PMC2797085

[39] Barabasi AL Gulbahce N Loscalzo J. Network medicine: a network-based approach to human disease. Nat Rev Genet (2011) 12:56–68. 10.1038/nrg2918 21164525 PMC3140052

[40] Hu CD Chinenov Y Kerppola TK. Visualization of interactions among bZIP and Rel family proteins in living cells using bimolecular fluorescence complementation. Mol Cell (2002) 9:789–98. 10.1016/S1097-2765(02)00496-3 11983170

[41] Citovsky V Lee LY Vyas S Glick E Chen MH Vainstein A Subcellular localization of interacting proteins by bimolecular fluorescence complementation in planta. J Mol Biol (2006) 362:1120–31. 10.1016/j.jmb.2006.08.017 16949607

[42] Morell M Espargaro A Aviles FX Ventura S. Detection of transient protein-protein interactions by bimolecular fluorescence complementation: the Abl-SH3 case. Proteomics (2007) 7:1023–36. 10.1002/pmic.200600966 17352427

[43] Silva NM Vieira JC Carneiro CM Tafuri WL. *Toxoplasma gondii*: the role of IFN-gamma, TNFRp55 and iNOS in inflammatory changes during infection. Exp Parasitol (2009) 123:65–72. 10.1016/j.exppara.2009.05.011 19501090

[44] Suzuki Y Conley FK Remington JS Importance of endogenous IFN-gamma for prevention of toxoplasmic encephalitis in mice. J Immunol (1950) 1989(143):2045–50. 2506275

[45] Yarovinsky F. Innate immunity to *Toxoplasma gondii* infection. Nat Rev Immunol (2014) 14:109–21. 10.1038/nri3598 24457485

[46] Mordue DG Sibley LD. A novel population of Gr-1+-activated macrophages induced during acute toxoplasmosis. J Leukoc Biol (2003) 74:1015–25. 10.1189/jlb.0403164 12972511

[47] Bliss SK Butcher BA Denkers EY Rapid recruitment of neutrophils containing prestored IL-12 during microbial infection. J Immunol (1950) 2000(165):4515–21. 10.4049/jimmunol.165.8.4515 11035091

[48] Del Rio L Bennouna S Salinas J Denkers EY CXCR2 deficiency confers impaired neutrophil recruitment and increased susceptibility during *Toxoplasma gondii* infection. J Immunol (1950) 2001(167):6503–9. 10.4049/jimmunol.167.11.6503 11714818

[49] Liu CH Fan YT Dias A Esper L Corn RA Bafica A Cutting edge: dendritic cells are essential for in vivo IL-12 production and development of resistance against *Toxoplasma gondii* infection in mice. J Immunol (1950) 2006(177):31–5. 10.4049/jimmunol.177.1.31 16785494

[50] Wheway J Herzog H Mackay F NPY and receptors in immune and inflammatory diseases. Curr Top Med Chem (2007) 7:1743–52. 10.2174/156802607782341046 17979783

[51] Barrionuevo P Beigier-Bompadre M Ilarregui JM Toscano MA Bianco GA Isturiz MA A novel function for galectin-1 at the crossroad of innate and adaptive immunity: galectin-1 regulates monocyte/macrophage physiology through a nonapoptotic ERK-dependent pathway. J Immunol (1950) 2007(178):436–45. 10.4049/jimmunol.178.1.436 17182582

[52] Levroney EL Aguilar HC Fulcher JA Kohatsu L Pace KE Pang M Novel innate immune functions for galectin-1: galectin-1 inhibits cell fusion by Nipah virus envelope glycoproteins and augments dendritic cell secretion of proinflammatory cytokines. J Immunol (1950) 2005(175):413–20. 10.4049/jimmunol.175.1.413 PMC4428613 15972675

[53] Foell D Wittkowski H Kessel C Luken A Weinhage T Varga G Proinflammatory S100A12 can activate human monocytes via toll-like receptor 4. Am J Respir Crit Care Med (2013) 187:1324–34. 10.1164/rccm.201209-1602OC 23611140

[54] Chen M Zhou H Cheng N Qian F Ye RD. Serum amyloid A1 isoforms display different efficacy at toll-like receptor 2 and formyl peptide receptor 2. Immunobiology (2014) 219:916–23. 10.1016/j.imbio.2014.08.002 25154907 PMC4252704

[55] Zhong J Huang W Deng Q Wu M Jiang H Lin X Inhibition of TREM-1 and dectin-1 alleviates the severity of fungal keratitis by modulating innate immune responses. PLoS One (2016) 11:e0150114. 10.1371/journal.pone.0150114 26963514 PMC4786258

[56] Janeway CA Jr Travers P Shlomchik MJ Walport M Immunobiology: The Immune System in Health and Disease. 5th ed New York: Garland Science (2001).

[57] Bacci S Nakamura T Streilein JW. Failed antigen presentation after UVB radiation correlates with modifications of Langerhans cell cytoskeleton. J Invest Dermatol (1996) 107:838–43. 10.1111/1523-1747.ep12330994 8941671

[58] Kim MT Harty JT. Impact of inflammatory cytokines on effector and memory CD8+ T cells. Front Immunol (2014) 5:295. 10.3389/fimmu.2014.00295 24995011 PMC4062963

[59] Haque S Khan I Haque A Kasper L. Impairment of the cellular immune response in acute murine toxoplasmosis: regulation of interleukin 2 production and macrophage-mediated inhibitory effects. Infect Immun (1994) 62:2908–16. 8005679 10.1128/iai.62.7.2908-2916.1994 PMC302898

[60] Villegas EN Lieberman LA Carding SR Hunter CA. Susceptibility of interleukin-2-deficient mice to *Toxoplasma gondii* is associated with a defect in the production of gamma interferon. Infect Immun (2002) 70:4757–61. 10.1128/IAI.70.9.4757-4761.2002 12183516 PMC128219

[61] Hwang S Khan IA. CD8+ T cell immunity in an encephalitis model of *Toxoplasma gondii* infection. Semin Immunopathol (2015) 37:271–9. 10.1007/s00281-015-0483-7 25944514 PMC6748331

[62] Strack A Asensio VC Campbell IL Schluter D Deckert M. Chemokines are differentially expressed by astrocytes, microglia and inflammatory leukocytes in *Toxoplasma* encephalitis and critically regulated by interferon-gamma. Acta Neuropathol (2002) 103:458–68. 10.1007/s00401-001-0491-7 11935261

[63] Kikumura A Ishikawa T Norose K. Kinetic analysis of cytokines, chemokines, chemokine receptors and adhesion molecules in murine ocular toxoplasmosis. Br J Ophthalmol (2012) 96:1259–67. 10.1136/bjophthalmol-2012-301490 22790439

[64] Wen X Kudo T Payne L Wang X Rodgers L Suzuki Y Predominant interferon-gamma-mediated expression of CXCL9, CXCL10, and CCL5 proteins in the brain during chronic infection with Toxoplasma gondii in BALB/c mice resistant to development of toxoplasmic encephalitis. J Interferon Cytokine Res (2010) 30:653–60. 10.1089/jir.2009.0119 20626297 PMC2963637

[65] Davalos-Misslitz AC Rieckenberg J Willenzon S Worbs T Kremmer E Bernhardt G Generalized multi-organ autoimmunity in CCR7-deficient mice. Eur J Immunol (2007) 37:613–22. 10.1002/eji.200636656 17304629

[66] Menning A Hopken UE Siegmund K Lipp M Hamann A Huehn J. Distinctive role of CCR7 in migration and functional activity of naive- and effector/memory-like Treg subsets. Eur J Immunol (2007) 37:1575–83. 10.1002/eji.200737201 17474155

[67] MartIn-Fontecha A Sebastiani S Hopken UE Uguccioni M Lipp M Lanzavecchia A Regulation of dendritic cell migration to the draining lymph node: impact on T lymphocyte traffic and priming. J Exp Med (2003) 198:615–21. 10.1084/jem.20030448 12925677 PMC2194169

[68] Semple F Dorin JR beta-Defensins: multifunctional modulators of infection, inflammation and more? J Innate Immun (2012) 4:337–48. 10.1159/000336619 22441423 PMC6784047

[69] Denkers EY Gazzinelli RT. Regulation and function of T-cell-mediated immunity during *Toxoplasma gondii* infection. Clin Microbiol Rev (1998) 11:569–88. 9767056 10.1128/cmr.11.4.569 PMC88897

[70] Sayos J Wu C Morra M Wang N Zhang X Allen D The X-linked lymphoproliferative-disease gene product SAP regulates signals induced through the co-receptor SLAM. Nature (1998) 395:462–9. 10.1038/26683 9774102

[71] Sharifi R Sinclair JC Gilmour KC Arkwright PD Kinnon C Thrasher AJ SAP mediates specific cytotoxic T-cell functions in X-linked lymphoproliferative disease. Blood (2004) 103:3821–7. 10.1182/blood-2003-09-3359 14726378

[72] Fleisher TA. Apoptosis. Ann Allergy Asthma Immunol (1997) 78:245–9; quiz 9–50. 10.1016/S1081-1206(10)63176-6 9087147

[73] Elmore S. Apoptosis: a review of programmed cell death. Toxicol Pathol (2007) 35:495–516. 10.1080/01926230701320337 17562483 PMC2117903

[74] Schaumburg F Hippe D Vutova P Luder CG. Pro- and anti-apoptotic activities of protozoan parasites. Parasitology (2006) 132(Suppl):S69–85. 10.1017/S0031182006000874 17018167

[75] Luder CG Gross U Lopes MF. Intracellular protozoan parasites and apoptosis: diverse strategies to modulate parasite-host interactions. Trends Parasitol (2001) 17:480–6. 10.1016/S1471-4922(01)02016-5 11587962

[76] Luder CG Gross U. Apoptosis and its modulation during infection with *Toxoplasma gondii*: molecular mechanisms and role in pathogenesis. Curr Top Microbiol Immunol (2005) 289:219–37. 10.1007/3-540-27320-4_10 15791958

[77] An HJ Shin H Jo SG Kim YJ Lee JO Paik SG The survival effect of mitochondrial Higd-1a is associated with suppression of cytochrome C release and prevention of caspase activation. Biochim Biophys Acta (2011) 1813:2088–98. 10.1016/j.bbamcr.2011.07.017 21856340

[78] Hou C Zhu M Sun M Lin Y. MicroRNA let-7i induced autophagy to protect T cell from apoptosis by targeting IGF1R. Biochem Biophys Res Commun (2014) 453:728–34. 10.1016/j.bbrc.2014.10.002 25305490

[79] Moubarak RS Planells-Ferrer L Urresti J Reix S Segura MF Carriba P FAIM-L is an IAP-binding protein that inhibits XIAP ubiquitinylation and protects from Fas-induced apoptosis. J Neurosci (2013) 33:19262–75. 10.1523/JNEUROSCI.2479-13.2013 24305822 PMC6618789

[80] Segura MF Sole C Pascual M Moubarak RS Perez-Garcia MJ Gozzelino R The long form of Fas apoptotic inhibitory molecule is expressed specifically in neurons and protects them against death receptor-triggered apoptosis. J Neurosci (2007) 27:11228–41. 10.1523/JNEUROSCI.3462-07.2007 17942717 PMC6673028

[81] Ron D Walter P. Signal integration in the endoplasmic reticulum unfolded protein response. Nat Rev Mol Cell Biol (2007) 8:519–29. 10.1038/nrm2199 17565364

[82] Wang T Zhou J Gan X Wang H Ding X Chen L *Toxoplasma gondii* induce apoptosis of neural stem cells via endoplasmic reticulum stress pathway. Parasitology (2014) 141:988–95. 10.1017/S0031182014000183 24612639

[83] Zhou J Gan X Wang Y Zhang X Ding X Chen L *Toxoplasma gondii* prevalent in China induce weaker apoptosis of neural stem cells C17.2 via endoplasmic reticulum stress (ERS) signaling pathways. Parasit Vectors (2015) 8:73. 10.1186/s13071-015-0670-3 25649541 PMC4322664

[84] Wan L Gong L Wang W An R Zheng M Jiang Z T. gondii rhoptry protein ROP18 induces apoptosis of neural cells via endoplasmic reticulum stress pathway. Parasit Vectors (2015) 8:554. 10.1186/s13071-015-1103-z 26489755 PMC4618732

[85] Dai RY Chen Y Fu J Dong LW Ren YB Yang GZ p28GANK inhibits endoplasmic reticulum stress-induced cell death via enhancement of the endoplasmic reticulum adaptive capacity. Cell Res (2009) 19:1243–57. 10.1038/cr.2009.104 19736567

