# Supplementary figures and images for: Genome-Wide Bimolecular Fluorescence Complementation-Based Proteomic Analysis of Toxoplasma gondii ROP18’s Human Interactome Shows Its Key Role in Regulation of Cell Immunity and Apoptosis
Source: Front Immunol. 2018 Feb 5;9:61. doi: 10.3389/fimmu.2018.00061 (PMC5807661; doi:10.3389/fimmu.2018.00061)

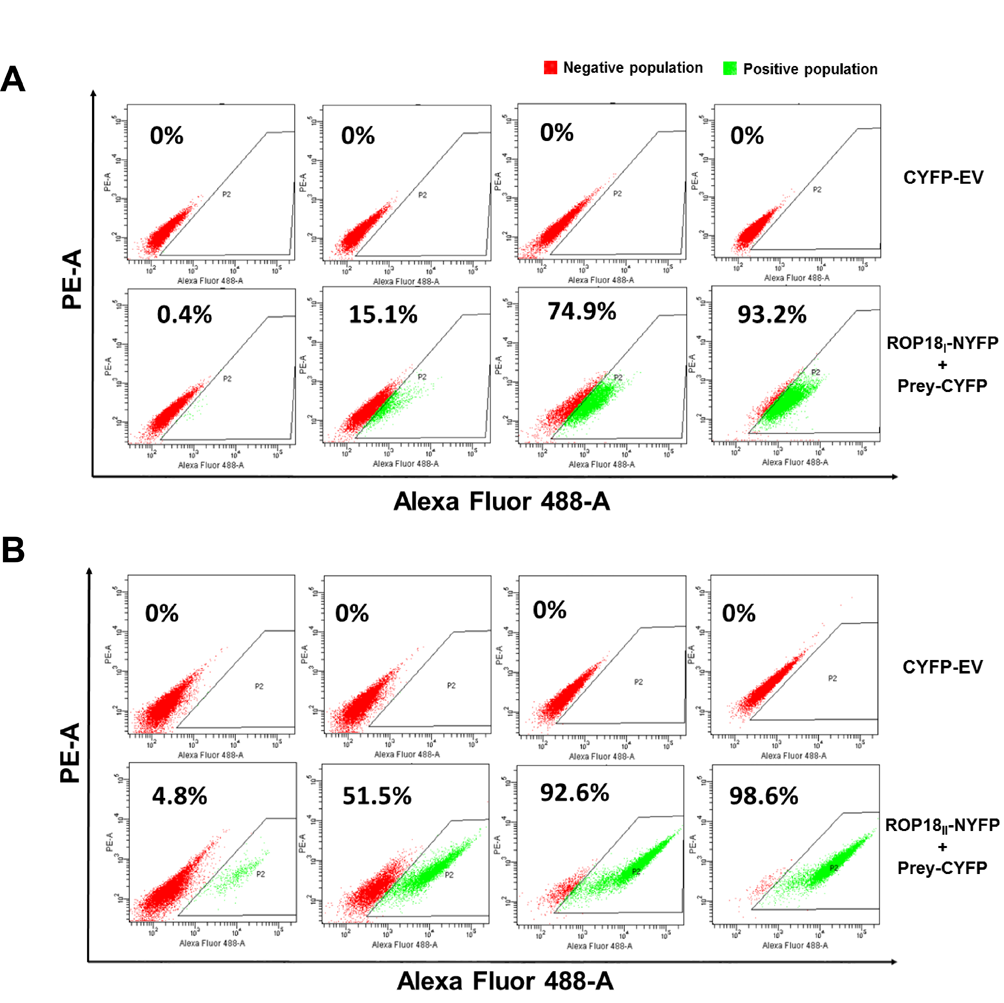

Supplement: Figure S1 — Flow cytometry histograms of HTC75 cells co-expressing ROP18I- (upper panel) or ROP18II-NYFP (lower panel) and Prey-CYFP, or expressing control constructs CYFP-EV, showing the ultimate positive sorting rate was more than 90%. EV, empty vector. [file Image_1.tif]
